# Medicine Men of the World

**Published:** 1888-01-28

**Authors:** 


*(To be continued.)*

### MEDICINE MEN OF THE WORLD.

BY THE MAN IN THE CROWD.

### VI.—SAVAGE AND CIVILISED PRACTITIONERS.

(*Continued from p.* 184.)

IN an earlier article it was observed that in most parts of the world the way into the medical profession was beset by difficulties, greater or less. China would appear to be an exception to this rule, if some who profess to know all about it may be relied upon. That "if" is, no doubt, an important one. A good deal that is written about China should be received *cum grano*, but it may be accepted as a fact that any Chinaman who thinks he has a talent for medicine is at full liberty to set up as a doctor. If he thinks it expedient to study one or two books on the subject of physiology or materia medica, he may, of course, do so, but nobody in China has any right to require it of him if he prefers to make a beginning without any such preparation. A few technical terms and a little learned jargon are, indeed, indispensable to the acquisition of the confidence of his patients, but he may, if he can, pick these up without books at all, or without any sort of medical training. The official theory appears to be that every man has so great an interest in his own health that he is not likely to patronise medical pretenders. The vast territory of China, therefore, appears to be a happy hunting-ground for quacks and impostors in the medical line.

And yet not altogether happy either. For, though the Government will do nothing by way of giving its imprimatur to those whom it considers well-qualified practitioners, it may come down very heavily on the unlucky Esculap whose patient dies under his hand. The Chinese Penal Code lays it down that—"When those who shall exercise the profession of medicine or surgery without understanding them"—[Oh, alarming comprehensiveness!]—"and shall administer drugs, or operate with a piercing or cutting instrument in a manner contrary to established rules and practice, and such that they shall thereby contribute to cause the death of the patient," the magistrates shall empanel a jury of other medical men, and if they find he has acted with evil intention, he may be convicted of manslaughter. If he has only acted in error, he may get off the punishment of manslaughter by paying the fine usual in cases of accidental killing; but he is to be compelled to quit the profession for ever. This certainly looks drastic enough, but Chinese Executive usually lags a good way behind the virtuous intention of the Legislature, and notwithstanding these stringent provisions the medical profession of China is said to be almost entirely composed of quacks and priests. People in China take to doctoring as people in England used at one time to take to schoolkeeping—because everybody was free to do it, and no special qualifications were required.

Mr. C. H. Eden, in an interesting book on the Chinese, quotes various authorities on Chinese medicine and medicine men. One of these authorities, the Abbé Huc, says: "It is very much the custom not to pay for medicines that have not produced a good effect, which happens pretty often; and even this is not the worst of the poor doctor's case. He is not unfrequently obliged to hide himself or fly the country to avoid imprisonment. This may happen when, having promised to cure a patient, he may be so awkward as to allow him to die. The relatives then, without hesitation, commence a lawsuit against him, and the safest way, if the doctor have a regard for his life or his pocket, is to take flight." Even under the best of circumstances, the Chinese doctor clearly has an uphill course. His visits, as a rule, are not paid for. He has to make what he can out of the drugs he supplies, and for these unlimited competition compels him to give long credit.

Pēre Du Halde tells us that the whole science of the Chinese physician consists in the knowledge of the pulse and the use of simples, of which they have a great variety, and which, according to them, have specific virtues to cure divers distempers. The study of the pulse is the great feature of their diagnosis, and some idea of the profundity of John Chinaman's medical wisdom may be gathered from what Du Halde says upon this point: "It is the knowledge and perfect comprehension of these beatings and percussions which discover the dispositions of the body and the affections which they receive from the elements; 'tis by these beatings that one may know the nature of the blood and spirit, as likewise what defects and excesses may be found therein; and it is the part of a skilful physician to regulate and reduce them to their first temperament." There are about a dozen pulses about the body known to the Chinese, and the great art of the doctor is to grope his way about these till he has tracked the disease to its lair. "They pretend to know"—what Du Halde evidently means is that they profess to know—"by the beating of the pulse only what is the cause of the disease, and in what part of the body it resides." They tell the patient in what part of the body the pain lies, and whether the head, stomach, or belly, or whether it be the liver or spleen which is affected; they likewise foretell when his head will be easier, when he shall recover his stomach, and when the distemper will leave him. "I speak," continues the writer, "of skilful physicians, and not of a sort of people who profess the art merely to get a livelihood without either study or experience; but it is certain—and there is no room to doubt all the testimonies we have—that the Chinese physicians have acquired a knowledge in this matter which is very extraordinary and surprising."

John Chinaman has, of course, the profoundest contempt for European doctors, and rarely or never consults them. "He prefers to appear," says Mr. Giles, a member of our China Consular service, who has written some interesting sketches of Chinese life, "with large dabs of green plaster stuck on either temple, and to drink loathsome decoctions of marvellous drugs, compounded according to eternal principles laid down many centuries ago. Directly a foreigner comes upon the scene, they seem to forget at once that medicine is an uncertain science, and expect not only a sure, but an almost instantaneous recovery; and, unfortunately a single failure is quite enough to undo the good of many months of successful practice." One Chinaman, the writer goes on to narrate, complained bitterly of a foreign doctor, and sweepingly denounced the whole system of Western medicine, because the practitioner alluded to had failed to cure his mother, who was eighty years old, and had had a severe paralytic seizure.

If, however, Europeans are often given to such pranks as that played off on one unlucky Celestial, who was ill-advised enough to apply to a Western resident for something to relieve a sick headache, it can hardly be thought surprising that European doctoring makes little headway in China. On the application of the sufferer alluded to, "our friend immediately bethought himself of a seidlitz powder; but when all was ready—the acid in one glass of water, and the salt in another—the devil entered into him, and he gave them to his victim to drink one after the other." The result was indescribable, for the mixture fizzed inside, and the unfortunate coolie passed such a *mauvais quart d'heure* as effectually to cure his experimenting master from any further indulgence in practical jokes of so extremely dangerous a nature.

